# Cautious use of activated prothrombin complex concentrate in patients receiving emicizumab: new evidence and clinical considerations

**DOI:** 10.1016/j.rpth.2025.102983

**Published:** 2025-07-24

**Authors:** Cedric Hermans

**Affiliations:** Division of Hematology, Hemostasis and Thrombosis Unit, Saint-Luc University Hospital, Université catholique de Louvain (UCLouvain), Brussels, Belgium

Emicizumab (EMI), a bispecific monoclonal antibody that mimics the function of activated factor (F)VIII and is not recognized by anti-FVIII antibodies, has established itself as the prophylactic treatment of choice for people with hemophilia A, both with and without persistent inhibitors [[1], [2], [3]]. However, EMI only partially restores coagulation. Despite its high efficacy in preventing bleeding episodes, it does not provide complete protection and cannot be considered a standalone treatment [4]. Adjunctive hemostatic therapy is required in patients on EMI who experience breakthrough bleeds or undergo major invasive procedures [5]. In non-inhibitor patients, supplementary FVIII administration remains necessary, whereas in patients with inhibitor, additional bypassing agents (BPAs), most notably activated prothrombin complex concentrate (APCC) or recombinant activated FVII (rFVIIa), are required to manage bleeding events or surgical interventions.

Safety concerns about the concomitant use of APCC with EMI emerged following the report of thrombotic events and thrombotic microangiopathy (TMA) during the HAVEN-1 trial [1]. These adverse events were observed in the context of high cumulative doses of APCC (> 100 U/kg/d) administered over prolonged periods (>24 hours). This can readily be explained by the high concentrations of activated clotting factors, particularly activated FIX and FX, present in APCC, which can bind with high affinity to EMI, potentially leading to excessive thrombin generation and, in some instances, TMA. In response, a warning was included in the United States product label against the use of APCC at cumulative doses exceeding 100 U/kg/d for >24 hours [6]. This caution also influenced clinical guidelines, which preferentially recommend the use of rFVIIa over APCC for the management of breakthrough bleeding and major surgical interventions in patients receiving EMI [7].

Consequently, rFVIIa has become the preferred adjunctive hemostatic agent in patients treated with EMI. However, rFVIIa is not universally effective and may fail to achieve optimal hemostasis in certain patients or specific bleeding episodes. Although both APCC and rFVIIa have demonstrated comparable efficacy and safety profiles when administered according to guideline-recommended dosing, inter-individual variability in treatment response remains a significant clinical challenge [8].

Prior to the widespread adoption of EMI, most treatment centers routinely maintained access to both classes of BPAs and tailored therapy based on each patient’s clinical response. In cases where monotherapy with either rFVIIa or APCC proved insufficient, clinicians often employed sequential or combined use of BPAs, with careful clinical and laboratory monitoring given the associated thrombotic risk [9]. The exclusion of APCC from the therapeutic arsenal has, in this context, narrowed the range of available options, particularly for managing difficult-to-treat bleeding episodes.

In this context, reevaluating the role of APCC in patients receiving EMI, especially with regards to dose, duration, and indication, is both timely and necessary. This question was recently addressed by Sidonio et al. [10], who reviewed available clinical trials data and real-world evidence on the efficacy and safety of APCC in EMI-treated patients. In total, 153 instances of APCC use were identified, 146 from clinical trials and 7 from real-world evidence, involving 51 patients receiving EMI prophylaxis (47 for bleeding episodes and 4 for surgical procedures), among whom 2 thrombotic events and 5 cases of TMA were reported. All events occurred in patients who had received APCC at doses exceeding 100 U/kg/d for ≥24 hours. Notably, 4 of the 5 TMAs occurred in patients who had also received concomitant rFVIIa for the same bleeding episode.

Based on these findings, this article proposes a shift in the clinical management of bleeding in EMI-treated patients by advocating for the cautious reintroduction of APCC. When its use is deemed necessary, APCC should be administered at doses no greater than 100 U/kg/d. The concomitant use of APCC and rFVIIa should be avoided due to the potential for cumulative thrombin generation and an increased risk of thrombosis. Close clinical and, where available, laboratory monitoring is essential for all patients receiving BPAs in the context of EMI prophylaxis, particularly those with additional thrombotic risk factors.

The high-quality review and analysis conducted by Sidonio et al. [10] provides valuable updated insights into the use of APCC in EMI-treated patients. However, several important limitations must be carefully considered when translating these observations and recommendations into clinical practice.

First, the review is narrative in nature and subject to inherent methodologic limitations. The clinical trial populations included in the analysis consisted of carefully selected and closely monitored individuals, whereas real-world data are both limited and inherently more heterogeneous. Moreover, the potential underreporting of adverse outcomes in real-world settings may introduce reporting bias and contribute to an overly optimistic assessment of APCC safety.

Another relevant limitation is the absence of individual patient-level data, which prevents any meaningful assessment of potential unrecognized determinants or risk factors for thrombotic complications. As acknowledged by the authors, no consistent clinical features were identified among patients who experienced thrombotic events, except for 2 recurring factors: the concomitant use of BPAs in 4 of 5 cases of TMA and the administration of APCC at doses exceeding 100 U/kg/d for ≥24 hours in all reported cases of thrombosis or TMA.

A further limitation is the relatively small number of reported thrombotic events. While this low incidence is somewhat reassuring, it significantly limits the statistical power to detect meaningful associations between APCC exposure and thrombotic risk, particularly at lower doses. Additionally, most available data pertain to breakthrough bleeding episodes in patients receiving EMI prophylaxis, whereas data on the perioperative use of APCC remain limited.

The review focuses predominantly on EMI, reflecting the scarcity of clinical data for patients treated with other nonfactor therapies and the complete absence of data for newer generations of FVIII mimetics currently in development or early clinical use. Consequently, the observations and recommendations presented in this analysis cannot be extrapolated to next-generations subcutaneously or orally administered FVIII mimetics, which will require independent and rigorous efficacy and safety assessments.

The potential role of antifibrinolytic agents, particularly tranexamic acid, in modulating thrombotic risk when used in conjunction with BPAs could not be explored in the review. Finally, the applicability of the recommendations should be limited to patients with congenital hemophilia. They should not be extended to individuals with acquired hemophilia, a population in which EMI is increasingly being used, but which often present with multiple concurrent thrombotic risk factors [11]. Further rigorous studies are warranted to assess the safety and efficacy of low-dose APCC in patients with acquired hemophilia who are receiving EMI.

Beyond these limitations, it remains difficult to anticipate the impact of this study’s conclusions and the extent to which its recommendations will be implemented in routine clinical practice. Multiple initiatives have previously aimed to raise awareness of the thrombotic risks associated with the concomitant use of EMI and APCC, strongly advocating against their combined administration. Although the reported cases of thrombosis have been rare, they have had an important effect on the perceived safety profile of EMI, even among patients not co-treated with APCC [12]. Whether the new findings of this study will be sufficient to restore confidence and prompt clinicians to reconsider the broader use of APCC remains uncertain. Furthermore, it is unclear how regulatory agencies will interpret these new data or how they may shape future guidelines and clinical recommendations. Importantly, these findings, along with their limitations, should be shared with patient organizations and advocacy groups and integrated into the shared decision-making process for any patient on EMI considered for cotreatment with APCC. From a practical perspective, the use of APCC has already declined significantly, and in some regions, the product may no longer be readily accessible. If APCC is to be reintroduced in this context, the recommendations proposed in this review should be followed rigorously. Finally, and now more than ever, this complex cotreatment must be overseen by experienced clinicians who are equipped to carefully assess the individual risk–benefit profile and to ensure thorough documentation of each case.
